# XIAP gene expression and function is regulated by autocrine and paracrine TGF-β signaling

**DOI:** 10.1186/1476-4598-9-216

**Published:** 2010-08-16

**Authors:** Céline Van Themsche, Parvesh Chaudhry, Valérie Leblanc, Sophie Parent, Eric Asselin

**Affiliations:** 1 Research group in Molecular Oncology and Endocrinology, Department of Chemistry-Biology, University of Quebec at Trois-Rivieres, Trois-Rivières, Quebec, Canada; 2 Department of Chemistry-Biology, University of Quebec at Trois-Rivières, 3351, boul. Des Forges, CP 500, Trois-Rivieres, Quebec, Canadaé

## Abstract

**Background:**

X-linked inhibitor of apoptosis protein (XIAP) is often overexpressed in cancer cells, where it plays a key role in survival and also promotes invasiveness. To date however, the extracellular signals and intracellular pathways regulating its expression and activity remain incompletely understood. We have previously showed that exposure to each of the three TGF-β (transforming growth factor beta) isoforms upregulates XIAP protein content in endometrial carcinoma cells *in vitro*. In the present study, we have investigated the clinical relevance of TGF-β isoforms in endometrial tumours and the mechanisms through which TGF-β isoforms regulate XIAP content in uterine cancer cells.

**Methods:**

TGF-β isoforms immunoreactivity in clinical samples from endometrial tumours was assessed using immunofluorescence. Two model cancer cell lines (KLE endometrial carcinoma cells and HeLa cervical cancer cells) and pharmacological inhibitors were used to investigate the signalling pathways regulating XIAP expression and activity in response to autocrine and paracrine TGF-β in cancer cell.

**Results:**

We have found immunoreactivity for each TGF-β isoform in clinical samples from endometrial tumours, localizing to both stromal and epithelial/cancer cells. Blockade of autocrine TGF-β signaling in KLE endometrial carcinoma cells and HeLa cervical cancer cells reduced endogenous XIAP mRNA and protein levels. In addition, each TGF-β isoform upregulated XIAP gene expression when given exogenously, in a Smad/NF-κB dependent manner. This resulted in increased polyubiquitination of PTEN (phosphatase and tensin homolog on chromosome ten), a newly identified substrate for XIAP E3 ligase activity, and in a XIAP-dependent decrease of PTEN protein levels. Although each TGF-β isoform decreased PTEN content in a XIAP- and a Smad-dependent manner, decrease of PTEN levels in response to only one isoform, TGF-β3, was blocked by PI3-K inhibitor LY294002.

**Conclusions:**

XIAP gene expression and function is positively regulated by exposure to the three TGF-β isoforms in a Smad-dependent manner, similar to constitutive XIAP gene expression which depends on autocrine TGF-β/Smad signalling.

## Background

TGF-β (transforming growth factor-beta) is a major regulator of proliferation, survival, migration/invasion and metastasis in cancer cells (reviewed in [1]). Upon ligand binding, TGF-β receptor I (TGFβ-RI) recruits and phosphorylates Smad2 and Smad3: phosphorylated Smad2 or Smad3 then associate with Smad4 to form heterodimeric complexes that translocate to the nucleus, where they can trigger downstream transcriptional responses [2]. Apart from this canonical Smad signalling pathway, TGF-beta can also activate ERK [3] and PI3-K [4] pathways. Most data concerning TGF-β signaling and function comes from studies focusing on TGF-β1. However, three TGF-β isoforms have been identified in mammalian cells: TGF-β1, TGF-β2 and TGF-β3. The three TGF-β isoforms can play redundant roles in cancer cells. However, recent studies have shown that TGF-β isoforms can differentially regulate cancer cell phenotype: in prostate cancer cells for example, TGF-β2, but not TGF-β1, confers resistance to TNFα-induced apoptosis [5]. Similarly, TGF-β3, but not TGF-β1 or TGF-β2, increase the invasiveness of endometrial carcinoma cells in vitro [6].

XIAP (X-linked inhibitor of apoptosis protein) plays a key antiapoptotic role in endometrial carcinoma cells. This member of the inhibitor of apoptosis protein family can directly inhibit caspases-3, -7, and -9 [6], and we recently observed that XIAP protects endometrial carcinoma cells against various proapoptotic agents, including TGF-β [6], TNFα [7] and chemotherapeutic drugs [8]. We have recently reported that exposure to each of the three TGF-β isoforms increase XIAP protein levels in endometrial carcinoma cells [6]. Our results suggested that TGF-β isoforms differentially activate intracellular signaling pathways in endometrial carcinoma cell: indeed, only TGF-β3 activates PI3-K/Akt pathway and increases XIAP protein levels in a PI3-K-dependent manner in these cells [6]. The different molecular mechanisms through which each TGF-β isoform increases XIAP protein content thus remains to be determined.

We have recently highlighted a new function for XIAP in cancer cells, in promoting polyubiquitination and proteasomal degradation of PTEN (phosphatase and tensin homolog deleted on chromosome ten) [9]. PTEN is a critical tumour suppressor [10], which negatively regulates pro-survival PI3-K/Akt pathway through its lipid phosphatase activity [11], and inhibits several regulators of cell cycle progression, including MAPK superfamily member ERK, through its protein phosphatase activity [12]. XIAP-induced degradation of PTEN is thus one of the mechanisms through which cancer cells can achieve successful inactivation of PTEN tumour suppressor function. Cellular factors regulating XIAP-induced degradation of PTEN, however, remain to be identified. We have showed that TGF-β3 induces XIAP-dependent degradation of PTEN [9]: since TGF-β1 and TGF-β2 also increase XIAP levels in cancer cells, but through mechanisms different from TGF-β3 [6], we hypothesized that, compared to TGF-β3, these isoforms would differently regulate XIAP-induced degradation of PTEN.

In the present study, we have used KLE endometrial carcinoma cell line and HeLa cervical cancer cell line, a widespread model for the study of cancer cell signaling [13,14], to determine the molecular mechanisms responsible for the upregulation of XIAP by each TGF-β isoform, as well as the consequence on XIAP-induced degradation of PTEN. We have found that autocrine TGF-β signalling as well as exposure to exogenous TGF-β isoforms upregulate XIAP expression at the transcriptional level, in a Smad/NF-κB dependent manner, and promote XIAP-induced proteasomal degradation of PTEN.

## Results

**Figure 1:**
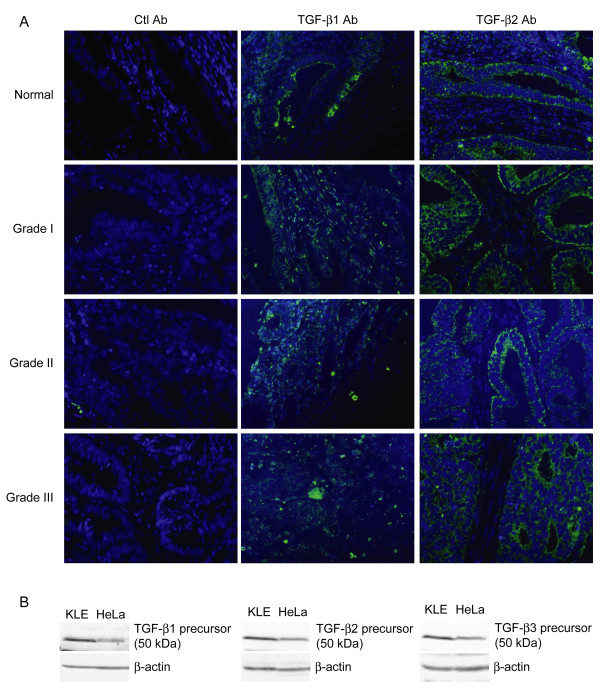
**The three TGF-β isoforms are present in human endometrial tumours**. A) TGF-β1 and TGF-β2 immunoreactivity (green) was determined in human endometrial carcinoma tissues grade I, II and III and normal endometrial tissue, using Cybrdi human endometrial tissue array slides. Tissues were obtained by biopsies. Negative control staining was obtained by replacing primary rabbit anti-TGF-βantibodies with normal rabbit antiserum. Hoechst dye was used to visualize nuclei (blue). Results shown are representative of 17 grade I tumour specimens, 33 grade II tumour specimens, 5 grade III tumour specimens and 3 normal endometrial specimens. Magnification: 400×. B) Autocrine synthesis of each TGF-β isoform was determined in KLE endometrial carcinoma cells and HeLa cervical cancer cells using western blot. β-actin was used as a loading control.

*The three TGF-β isoforms are present in human endometrial tumours*. We have previously shown that TGF-β3 immunoreactivity can be detected in clinical samples from endometrial carcinoma patients [6]. In the present study, we have found the presence of TGF-β1 and TGF-β2 immunoreactivity in these clinical samples (Figure 1A), indicating that each TGF-β isoform is present in the tumour microenvironment. Contrary to TGF-β3 immunoreactivity, which was detectable in normal as well as grade I and grade II samples but not in grade III samples [6], TGF-β1 and TGF-β2 immunoreactivity was detectable throughout cancer progression, even in grade III tumours (Figure 1A). Similar to TGF-β3 [6], TGF-β1 and TGF-β2 immunoreactivity was detectable in both epithelial and stromal compartments of endometrial tumours (Figure 1A), suggesting that both autocrine and paracrine TGF-β signalling takes place in these tumours. The hypothesis of autocrine TGF-β signaling in endometrial tumours is strengthened by the observation that endometrial carcinoma cell lines such as KLE constitutively produces the precursor protein of all three TGF-β isoforms *in vitro *(Figure 1B). Similar to KLE cells, HeLa cervical cancer cells constitutively produced precursor protein for each TGF-β isoform (Figure 1B), indicating that production of more than one TGF-β isoform is not a unique feature of endometrial cancer cells.

**Figure 2:**
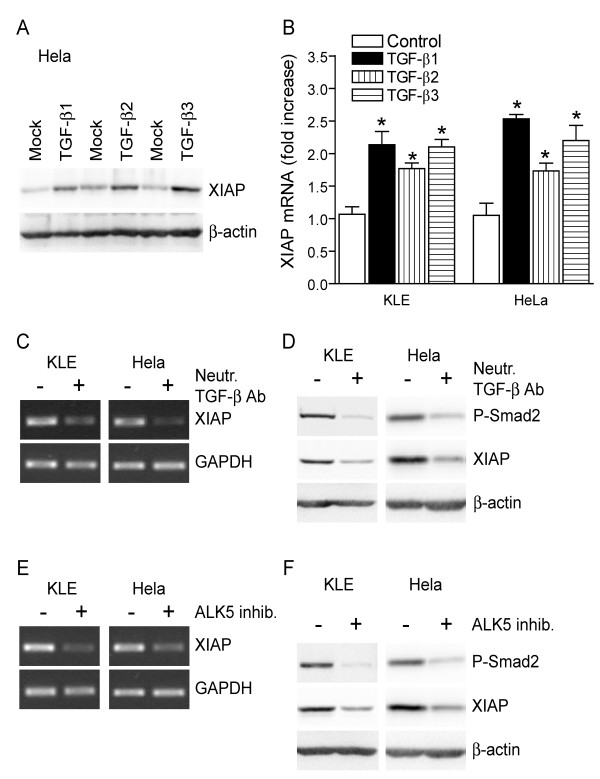
**Autocrine and paracrine TGF-β signaling regulates XIAP gene expression in a Smad-dependent manner**. A-B) HeLa human cervical cancer cell line and KLE human endometrial carcinoma cell line were treated with the indicated recombinant TGF-β isoforms (10 ng/mL) or with vehicle, for 24 h. XIAP protein levels in treated HeLa cells were determined using western blot (A); XIAP mRNA levels in treated KLE and HeLa cells were determined using RT-PCR, and results from densitometric analysis are presented (B). C-D) KLE and HeLa cells were treated with 2 μg/mL anti-TGF-β neutralizing antibody or isotypic control antibody for 24 h, and XIAP mRNA levels were determined using RT-PCR (C) whereas XIAP protein levels were determined using western blot (D). E-F) KLE and HeLa cells were treated with 50 μM ALK5 inhibitor SB431543 or vehicle for 24 h, and XIAP mRNA levels were determined using RT-PCR (E) whereas XIAP protein levels were determined using western blot (F). In D,F), levels of phosphorylated Smad2 (P-Smad2) were determined to monitor the efficiency of TGF-β pathway inhibition. In all experiments, β-actin or GAPDH were used as loading controls. Graphs represent mean ± SE of three independent experiments. * p < 0.05 compared to control-treated cells.

*Autocrine and paracrine TGF-β signaling regulate XIAP gene expression*. We have previously reported that TGF-β isoforms increase XIAP protein levels in endometrial carcinoma cells [6] and we observed that each TGF-β isoform also upregulates XIAP protein content in HeLa cervical carcinoma cells (Figure 2A), indicating that the regulation of XIAP protein levels by TGF-β is not restricted to cancer cells from the endometrium. However, the mechanisms through which TGF-β isoforms regulate XIAP protein content in cancer cells remained unknown. In the present study, we have investigated these mechanisms. Given exogenously, each TGF-β isoform increased XIAP transcript levels (Figure 2B), revealing that paracrine TGF-β signaling regulates XIAP expression at the transcriptional level. In addition, blockade of autocrine TGF-β signaling (as showed by reduced levels of phosphorylated Smad2 (Figure 2D)) using neutralizing TGF-β antibody reduced endogenous XIAP transcript (Figure 2C) and protein (Figure 2D) levels. Similarly, treatment with ALK5 inhibitor SB431542, which blocked constitutive TGF-β receptor I kinase activity as shown by decreased levels of phosphorylated Smad2 (Figure 2F), also decreased XIAP transcript (Figure 2E) and protein (Figure 2F) levels. The latter results reveal that autocrine TGF-β signaling constitutively regulates XIAP gene expression.

**Figure 3:**
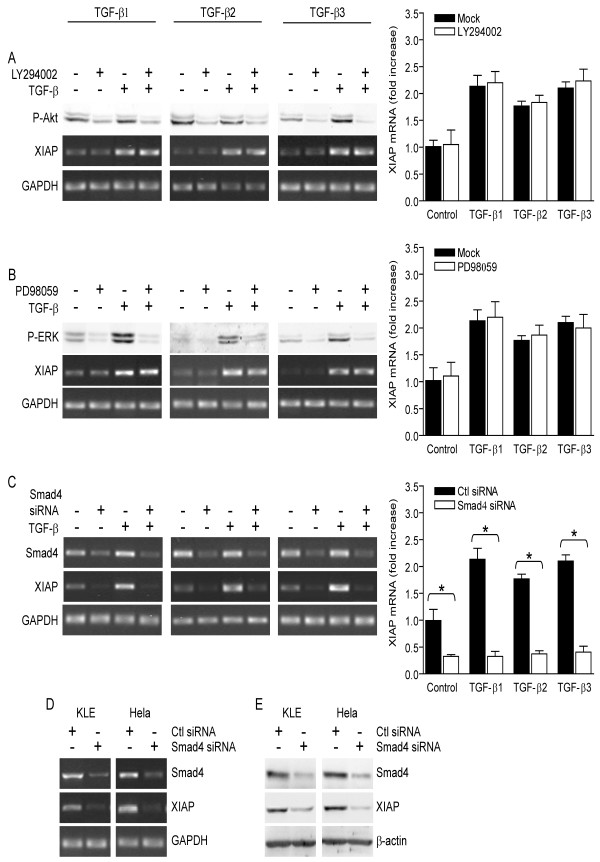
**TGF-β isoforms promote XIAP gene expression via Smad and PI3-K pathways**. A-C) Before KLE cells were treated with the indicated recombinant TGF-β isoforms (10 ng/mL) or with vehicle for 24 h, they were pre-treated with 50 μM PI3-K inhibitor LY294002 or vehicle for 1 h (A), 10 μM MEK1/ERK pathway inhibitor PD98059 or vehicle for 1 h (B) or Smad4 siRNA or control (scrambled) siRNA for 24 h (C). Levels of XIAP mRNA were determined using RT-PCR and results from densitometric analysis are presented. In all experiments, β-actin or GAPDH were used as loading controls. Levels of Smad4 protein, phosphorylated ERK (P-ERK) or phosphorylated Akt (P-Akt) were determined to monitor the efficiency of Smad4 RNAi, MEK/ERK pathway inhibition or PI3-K inhibition, respectively. Graphs represent mean ± SE of three independent experiments. *p < 0.05. D-E) KLE and HeLa cells were treated with control (scrambled) or Smad4 siRNA for 24 h and levels of XIAP mRNA (D) and protein (E) were determined using RT-PCR (D) and western blot analysis (E). β-actin and GAPDH were used as loading controls.

*TGF-β isoforms similarly promote XIAP gene expression via Smad pathway*. We have investigated the pathways mediating the upregulation of XIAP gene expression in response to each TGF-β isoform in KLE cells. PI3-K inhibitor LY294002 (Figure 3A) or ERK upstream kinase MEK1 inhibitor PD98059 (Figure 3B) did not inhibit the upregulation of XIAP mRNA in response to TGF-β isoforms, indicating that TGF-β-induced upregulation of XIAP gene expression is PI3-K- and ERK-independent. However, knockdown of Smad4 using RNAi blocked the upregulation of XIAP mRNA in response to each TGF-β isoform (Figure 3C), indicating that the upregulation of XIAP gene expression by exogenous TGF- isoforms is Smad-dependent. In addition, we found that knockdown of Smad4 using RNAi reduced endogenous levels of both XIAP mRNA (Figure3D) and protein (Figure 3E). Altogether, these results indicate that autocrine as well as paracrine TGF-β-induced signalling induces XIAP gene expression in a Smad-dependent manner.

**Figure 4:**
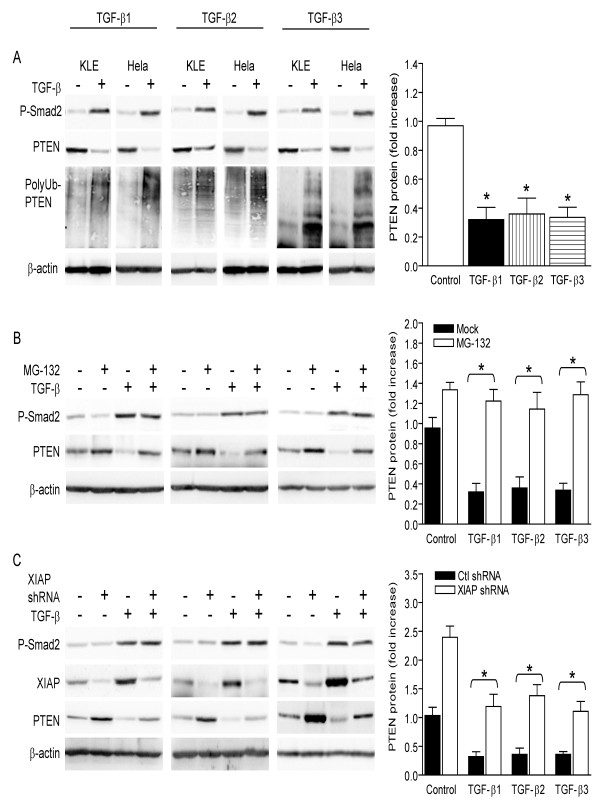
**TGF-β isoforms-induced decrease of PTEN protein content is XIAP-dependent**. A) KLE and HeLa cells were treated with the indicated recombinant TGF-β isoforms (10 ng/mL) or with vehicle for 24 h. Levels of phosphorylated Smad2 (P-Smad2) were determined to monitor the activation of Smad pathway by TGF-β isoforms. Levels of PTEN protein and its ubiquitination were determined using western blot. Graphs represent densitometrical analysis of PTEN in Hela Cells. B) HeLa cells were pre-treated with 10 μM proteasome inhibitor MG-132 for 1 h before they were treated with the indicated recombinant TGF-β isoforms (10 ng/mL) or with vehicle, for 24 h. Levels of PTEN protein were determined using western blot. C) Before treatment with the indicated recombinant TGF-β isoforms (10 ng/mL) or with vehicle for 24 h, HeLa cells were transfected with XIAP shRNA or control (scrambled) shRNA for 24 h. PTEN and XIAP protein levels were determined using western blot. In all experiments, levels of phosphorylated Smad2 (P-Smad2) were determined to monitor the activation of Smad pathway by TGF-β isoforms and β-actin was used as a loading control. Graphs represent mean ± SE of three independent experiments. * p < 0.05.

*TGF-β isoforms decrease PTEN protein content in a XIAP-dependent manner*. We have previously shown that overexpression of XIAP induces polyubiquitination and degradation of PTEN protein [9]. Therefore, we hypothesized that through their role in the regulation of XIAP gene expression (Figure 2-3), TGF-β isoforms regulate PTEN protein content in uterine carcinoma cells. In agreement with this, we found that upregulation of XIAP levels by each TGF-β isoform (Figure 2A) was accompanied by an increase of polyubiquitination of PTEN and a decrease of PTEN protein levels (Figure 4A). Pre-treatment of the cells with proteasome inhibitor MG-132 prevented TGF-β isoforms from decreasing PTEN protein content (Figure 4B), showing that TGF-β-induced decrease of PTEN involves proteasome activity. Further, we found that knockdown of XIAP using RNAi before exposure to each TGF-β isoform prevented TGF-β from decreasing PTEN protein levels (Figure 4C). Altogether, these results reveal that each TGF-β isoform negatively regulates PTEN content in uterine carcinoma cells, in a XIAP-dependent manner.

**Figure 5:**
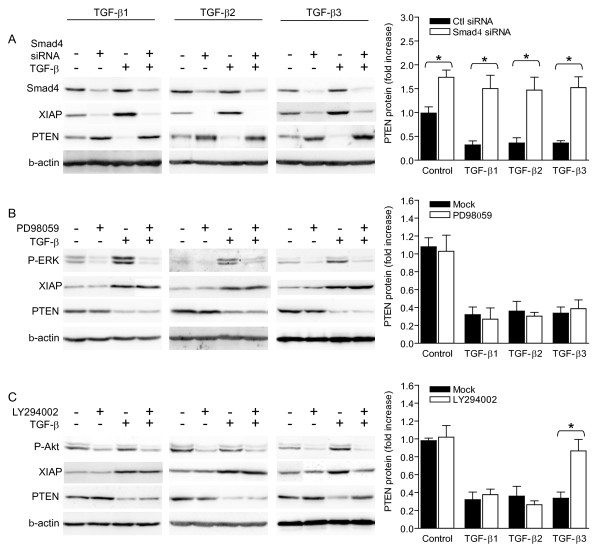
**TGF-β isoforms decrease PTEN protein content through differential pathways**. A-C) Before treatment with 10 ng/mL recombinant TGF-β isoforms or with vehicle for 24 h, KLE cells were pre-treated with Smad4 siRNA or control (scrambled) siRNA for 24 h (A), 10 μM MEK1/ERK pathway inhibitor PD98059 or vehicle for 1 h (B) or 50 μM PI3-K inhibitor LY294002 or vehicle for 1 h (C). Protein lysates used were identical to the ones used in figure 3. Levels of PTEN protein were determined using western blot. Levels of Smad4 protein, phosphorylated ERK (P-ERK) or phosphorylated Akt (P-Akt) were determined to monitor the efficiency of Smad4 RNAi, MEK/ERK pathway inhibition or PI3-K inhibition, respectively. In all experiments, β-actin was used as a loading control. Graphs represent mean ± SE of three independent experiments. * p < 0.05.

*TGF-β decreases PTEN protein content through isoform-specific pathways*. We have investigated the signaling pathways involved in downregulation of PTEN in response to the different TGF-β isoforms. Since Smad pathway is involved in the upregulation of XIAP gene expression by TGF-β isoforms (Figure 3) and that TGF-β regulates PTEN content in a XIAP-dependent manner (Figure 4), we first investigated whether TGF-β regulates PTEN content in a Smad-dependent manner. We found that interference with Smad4 RNA prevented each TGF-β isoform from decreasing PTEN protein content (Figure 5A). Then, blockade of ERK pathway activity using PD98059, resulting in decreased levels of phosphorylated ERK (P-ERK), had no impact on TGF-β-induced decrease of PTEN protein levels (Figure 5B). However, pharmacological inhibition of PI3-K activity, reflected by decreased levels of phosphorylated Akt (P-Akt), prevented TGF-β3-induced, but not TGF-β1- or TGF-β2-induced, reduction of PTEN protein content (Figure 5C). These results indicate that TGF-β decreases PTEN protein content in a Smad-dependent manner, but also through isoform-specific pathways as only TGF-β3 regulates PTEN content in a PI3-K-dependent manner.

**Figure 6:**
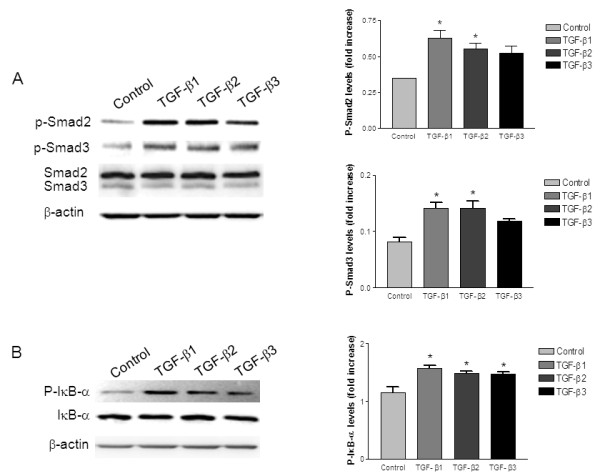
**TGF-β isoforms-induced phosphorylation/activation of Smad2, Smad3 and IκB-α**. HeLa and KLE cells (not shown) were treated with the indicated recombinant TGF-β isoforms (10 ng/mL) or with vehicle for 15 min. Levels of phosphorylated Smad2 (P-Smad2) and phosphorylated Smad3 (P-Smad3) were determined to monitor the activation of Smad pathway by TGF-β isoforms and levels of phosphorylated IκB-α was determined to examine the activation of NF-κB pathway. In all experiments, β-actin was used as a loading control. Graphs represent mean ± SE of three independent experiments. * p < 0.05 compared to control.

*Smad and NF-κB signaling pathway involvement in TGF-β mediated XIAP upregulation*. After verification of the TGF-β mediated XIAP upregulation and concomitant decrease in PTEN protein content, we investigated whether this signal is predominantly delivered via Smad-dependent and/or Smad-independent pathways. In Hela cells, TGF-β stimulation induced Smad2 and Smad3 phosphorylation. Total Smad2 and Smad3 levels were not modulated by TGF-β isoforms (Figure 6A). We also observed a similar increase in the phosphorylation/activation of Smad2 and Smad3 in KLE cells treated with each TGF-β isoforms (data not shown). It is known that IκB-α phosphorylation leads to activation, nuclear translocation and increase in transcriptional activity of NF-κB. In order to understand whether the XIAP upregulation is mediated through the activation of NF-κB by TGF-β isoforms, we performed western blot analysis with a phospho-specific antibody against IκB-α. TGF-β treatment resulted in rapid phosphorylation of IκB-α with no effect on total IκB-α levels (Figure 6B). Therefore, these results suggest that TGF-β induced XIAP upregulation is mediated through a TGF- β/Smad/NF-κB pathway.

## Discussion

In the past, most studies examining the role of TGF-β in cancer progression have focused on TGF-β1 isoform. However, a number of studies have shown that TGF-β2 and TGF-β3 are often expressed in human tumours [15,16]. Furthermore, the different TGF-β isoforms can sometimes differentially activate signaling pathways in cancer cells, leading to isoform-specific effects on cellular phenotype [5,6]. Dissecting the differential pathway activation and roles of TGF-β isoforms in cancer cells could foster the identification of specific factors regulating key aspects of tumour progression.

We have found that similar to various other cancer cell types [15,16], human endometrial tumours contain the three TGF-β isoforms. Since the proteins are detectable in both the epithelial and stromal counterparts of the tumours, they could be responsible for autocrine as well as paracrine signalling in the microenvironment of these tumours. We had previously shown that exposure to TGF-β isoforms increases XIAP protein content in endometrial carcinoma cells [6], and here we found that the three TGF-β isoforms upregulate XIAP expression, at the mRNA level, in these cells. TGF-β1 had previously been shown to increase XIAP gene expression [17], but the impact of TGF-β2 and TGF-β3 were unknown. Further, the present study revealed that autocrine TGF-β signaling constitutively promotes XIAP gene expression. To our knowledge, this is the first time a receptor-activated pathway responsible for endogenous production of XIAP by cancer cells is identified. RNAi has allowed us to determine that constitutive as well as exogenous TGF-β-induced XIAP gene expression involves Smad pathway. However, we have found no consensus sequence for Smad binding in the promoter of XIAP, suggesting that Smad transcription factors are not directly responsible for the induction of XIAP gene expression in response to TGF-β. It has been shown that Smad and NF-κB components interact and cooperate to regulate gene expression in response to TGF-β [18-20], and the role of NF-κB in constitutive expression of XIAP is well established [21]. In the present study we also found that upon TGF-β treatment both the components of Smad and NF-κB pathway are activated. Therefore, constitutive XIAP gene expression could be regulated via a TGF-β/Smad/NF-κB pathway.

The present study further demonstrates that regulation of XIAP expression by TGF-β isoforms impacts XIAP function in cancer cells, since each TGF-β isoform promotes XIAP-dependent degradation of PTEN when added exogenously. To produce this effect, the three TGF-β isoforms share a requirement for Smad signaling pathway, consistent with the observation that TGF-βs increase XIAP content via Smad pathway. However, decrease of PTEN protein levels in response to TGF-β3, but not TGF-β1 or TGF-β2, also requires PI3-K activity, in agreement with our observation that PI3-K activity is involved in TGF-β3, but not TGF-β1 or TGF-β2-induced upregulation of XIAP protein [6]. The reason why PI3-K activity is required, in addition to Smad signaling, for TGF-β3 to decrease PTEN protein levels is unknown. Since Akt has been shown to phosphorylate and stabilize XIAP protein [22], inhibition of PI3-K/Akt activity could be sufficient to reduce the stability of XIAP protein and its interaction with PTEN, leading to decreased ubiquitination and degradation of PTEN [9]. Alternatively, PI3-K activity has been shown to promote nuclear export of PTEN [23], which could favour interaction of PTEN with XIAP in the cytosol, thus promoting XIAP-induced degradation of PTEN. In fact, PI3-K and Smad pathways may interact to regulate TGF-β3-induced degradation of PTEN protein, since phosphorylated Akt interacts with Smad3 and prevents its phosphorylation and translocation to the nucleus [24,25]. In this scenario, balance between PI3-K and Smad pathway activities would regulate XIAP expression and XIAP-induced degradation of PTEN, and inhibition of one or the other pathway would be sufficient to block TGF-β3-induced decrease of PTEN protein levels. Above all, the fact that only TGF-β3 induces PI3-K-dependent decrease of PTEN protein levels highlights the isoform-specific nature of TGF-β-induced post-transcriptional regulation of PTEN content.

## Conclusions

The present study highlights the presence of the three TGF-β isoforms in clinical samples from endometrial carcinoma, and emphasizes the presence of autocrine TGF-β production and signaling in cancer cells. Autocrine TGF-β signaling constitutively regulates XIAP gene expression, in a Smad-dependent manner. Furthermore, exogenous/paracrine TGF-β signaling also transcriptionally upregulates XIAP content, in an isoform-specific manner. Finally, upregulation of XIAP in response to TGF-β regulates XIAP function on post-transcriptional regulation of PTEN protein content, and autocrine TGF-β signalling regulates compartmentalization of PTEN, probably in a XIAP-dependent manner. Altogether, these observations highlight a new role for TGF-β signaling in the regulation of XIAP gene expression and function.

## Methods

*Cell lines and reagents*. Human endometrial carcinoma cell line KLE and human cervical cancer cell line HeLa were purchased from ATCC. KLE cells were maintained in DMEM-F12 medium without HEPES supplemented with 10% FBS and 50 mg/mL gentamycin; HeLa cells were maintained in DMEM-F12 medium supplemented with 2% BGS and 50 mg/mL gentamycin. XIAP plasmid constructs were a kind gift from Dr. Robert G. Korneluk (University of Ottawa Eye Institute, Ottawa, Ont, Canada). All antibodies were from Cell Signaling Technology (Beverly, MA, USA) except for mouse monoclonal anti-actin antibody (Sigma, St-Louis, MO, USA), goat anti-rabbit, HRP-conjugated antibody (Bio-Rad Laboratories, Mississauga, ON, Canada), and anti-TGF-β antibodies (Santa Cruz Biotechnology, Santa Cruz, CA, USA). Recombinant TGF-βs were purchased from Calbiochem (San Diego, CA, USA). LY294002 and PD98059 were purchased from Cell Signaling Technology. SB431542 was purchased from Sigma.

*Immunofluorescence-based detection of TGF-β1 and TGF-β2 in clinical samples*. Preparation and image analysis was performed as previously described [6]. Specificity of anti-TGF-βantibodies had previously been confirmed by checkerboard peptide blocking experiments [26]. Briefly, the working dilution of each antibody (TGF-β1 (sc-146) and TGF-β2 (sc-90) from Santa Cruz Biotechnology) was incubated with a 10-fold excess of blocking peptide (Santa Cruz Biotechnology) overnight at 4°C before staining. In all cases, staining was abolished by homologous peptide but unaffected by pre-incubation with peptides corresponding to other isoforms [26].

*Cell treatments*. Cells were seeded in 6 well-plates at the required density to reach approximately 60% confluency after 24 h (0.2 × 10^6 ^KLE cells and 0.5 × 10^6 ^HeLa cells per well). The following day, medium was changed and replaced with fresh media containing the appropriate treatment.

*Western blots*. Equal amounts of total cell lysates or subcellular fractions (as determined using Bio-Rad DC protein assay) were separated onto 8-15% polyacrylamide gels and then transferred onto nitrocellulose membranes (Bio-Rad). The membranes were blocked with 5% milk in PBS-0.05% Tween-20 for 1 h at RT, probed with primary antibody (PTEN #9559; XIAP #2042; P-ERK #4377; P-Akt (Ser-473) #7291; Akt #9272; Smad3 #9513; Smad4 #9515; TGF-βRI #3712; all antibodies from Cell Signaling) overnight at 4°C, washed in PBS-0.05% Tween-20 and incubated with horseradish peroxidase-conjugated anti-rabbit secondary antibody (Bio-Rad). Detection was performed using SuperSignal West FemtoTM substrate (Pierce, Arlington Heights, IL, USA), as described by the manufacturer.

*RNA extraction and RT-PCR analysis*. Total RNA was isolated from cells using Trizol Reagent (Invitrogen, Burlington, ON, Canada) according to manufacturer's instructions. First strand cDNA was synthesized from 0.4 μg RNA using MMLV reverse transcriptase (Invitrogen). Primers for PCR amplification of XIAP were 5'-gagaagatgacttttaacagttttga-3' (sense) and 5'-ttttttgcttgaaagtaatgactgtgt-3' (antisense). Primers for amplification of PTEN were 5'-accaggaccagaggaaact-3' (sense) and 5'-gctagcctctggatttgacg-3' (antisense). Primers for amplification of Smad4 were 5'-gttgatggatacgtggaccc-3' (sense) and 5'-acctttgcctatgtgcaacc-3' (antisense). Primers for amplification of GAPDH were 5'-gtcagtggtggacctgacct-3' (sense) and 5'-tgagcttgacaaagtggtcg-3' (antisense). PCR reactions were conducted in a MJ Research Thermal cycler (model PTC-100), using the following parameters: 30 sec. at 94°C, 30 sec. at 58°C, and 1 min. at 72°C, for 35 cycles except for GAPDH (25 cycles). The reaction mixture was size-separated on an agarose gel and visualized using SYBR-SafeTM (Invitrogen) staining upon ultraviolet transillumination.

*Transfection with siRNAs*. Cells were seeded in 6-well plates at a required density to reach approximately 60% confluency in 24 h (0.2 × 10^6 ^KLE cells and 0.5 × 10^6 ^HeLA cells per well), and allowed to adhere overnight. The day of experiment, TGF-βRI (5'-ggacccuucauuagaucgctt-3' and 5'-gcgaucuaaugaagggucctc-3'), Smad4 (5'-ggucuuugauuugcgucagtt-3' and 5'-cugacgcaaaucaaagacctt-3') or control (5'-acucuaucugcacgcugacuu-3' and 5'-aagucagcgugcagauagagu-3') siRNAs were mixed with Mirus Trans-it TKO transfection reagent (Fisher Scientific, Ottawa, ONT, Canada) following supplier's instructions and added to the cells (100 nM working concentration). After 8 h-transfection, medium was replaced and plates were incubated for 16 additional hours (total: 24 h) or 40 additional hours (total: 48 h), as indicated in Figure legends, at 37°C before cells were collected.

*Transfection with shRNAs*. Cells were seeded in 6-well plates at the required density to reach approximately 60% confluency after 24 h. The day of transfection, XIAP shRNAs shRNA (5'-GCCACGCAGTCTACAAATTCT-3') or control (scrambled) shRNA (all shRNA inserted into pGeneClip (SABiosciences, Frederick, MD, USA)) were added to cells using a ratio of 3.6 μL Fugene:1.2 μg DNA/well. After 8 h-transfection, medium was replaced and plates were incubated for 40 additional hours (total: 48 h) at 37°C before cells were collected.

### Statistical analysis

Data were subjected to one-way ANOVA (PRISM software version 3.03; GraphPad, San Diego, CA). Differences between experimental groups were determined by the Tukey's test. Statistical significance was accepted when p < 0.05 and indicated as asterisk above individual graph bars.

## Competing interests

The authors declare that they have no Competing interests.

## Authors' contributions

CVT participated in design of the study, carried out the experiments, drafted and finalized writing of the manuscript. PC carried experiments and part of the writing. SP and VL participated in cell biology studies. EA participated in the design of the study and its writing. All authors read and approved the final manuscript.
